# Mental Health Literacy in Healthcare Students: An Expansion of the Mental Health Literacy Scale

**DOI:** 10.3390/ijerph17030948

**Published:** 2020-02-04

**Authors:** Hsing-Jung Chao, Yin-Ju Lien, Yu-Chen Kao, I-Chuan Tasi, Hui-Shin Lin, Yin-Yi Lien

**Affiliations:** 1 Department of Health Promotion and Health Education, National Taiwan Normal University, 162, Heping East Road Section 1, Taipei 106, Taiwan; 2 Department of Psychiatry, Tri-Service General Hospital Songshan Branch, Taipei 106, Taiwan

**Keywords:** mental health literacy, mental illness stigma, measurement, medical education, public health education, health professionals and students, Mental Health Literacy Scale for Healthcare Students (MHLS-HS)

## Abstract

Objective: Although the recently developed mental health literacy scale showed significant score differences between general population and mental health professionals, to this date there is no published scale intended to specifically assess mental health literacy (MHL) in healthcare students. This study constructed a 26-item scale-based measure to assess multiple components of MHL and associated psychometric properties in a sample of medical and public health students of 11 universities in Taiwan. Methods: The development and validation of the scale comprised three phases: measure development, pilot testing (n = 32), and psychometric properties examination (n = 1294). Results: 26 items were generated for five factors: maintenance of positive mental health, recognition of mental illness, attitude to mental illness stigma, help-seeking efficacy, and help-seeking attitude. The scale demonstrated good content validity, internal consistency, and construct validity (factorial validity, convergent validity, discriminant validity, and known groups validity). Conclusions: The findings suggest that the Mental Health Literacy Scale for Healthcare Students (MHLS-HS) is a valid, reliable, and practical tool for identifying MHL gaps in medical and public health students. It has the potential to inform remedial curricular interventions for educators and evaluate intervention effectiveness.

## 1. Introduction

The construct of mental health literacy (MHL) is still an evolving concept [1]. It originated from the concept of health literacy and was first introduced by Jorm and colleagues, who described it as “knowledge and beliefs about mental disorders which aid their recognition, management or prevention” [2]. A more recent definition has extended the previous definitions of MHL [3] to include four distinct but related components: (1) understanding how to obtain and maintain good mental health, (2) understanding mental disorders and their treatments, (3) reducing the stigma related to mental disorders, and (4) enhancing help-seeking efficacy (i.e., knowing when and where to obtain evidence-based mental health care and having the competencies to enhance self-care) [4,5,6]. This broader definition advances previous perceptions of MHL and includes not only knowledge and beliefs about mental ill-health, but also the promotion of mental health [1], in line with the World Health Organization’s definition of mental health [7]. Furthermore, the definition also includes the concept of stigma, which acts as a barrier to people seeking help for mental health problems and mental illness [8], as well as highlighting help-seeking efficacy as a key component of MHL [9]. Help-seeking efficacy is the key factor that influences help-seeking behavior; in addition, a help-seeking attitude is a strong predictor of help-seeking intention and behavior [10,11,12] and so should also be emphasized as a critical component of MHL [9].

Past research has often focused on improving the MHL of the general public [3,13,14]. However, many studies have indicated the need for effort to improve the MHL of health professionals and students, for several reasons. First, health professionals experience a high frequency of stress, burnouts, anxiety, or depression, which have deleterious effects on their physical and mental health, potentially resulting in a negative impact on quality of care [15,16,17,18,19,20,21]. Second, mental health problems and mental disorders often remain undiagnosed in the primary care setting [22,23], which may delay their early identification and the provision of appropriate intervention [3]. For example, it has been reported that emergency physicians might miss diagnosing up to two-thirds of patients with delirium in routine clinical observations [24]. Another study reported that the identification rate of common mental illnesses by physicians (i.e., hospital-based specialists, hospital-based family physicians, physicians in community health stations) was only 14% [25]. Additionally, the stigma about mental illnesses, which is widespread in the general public, may be shared by some health professionals and students. It has been shown that health professionals and students tend to hold negative attitudes or stereotypical beliefs toward people with mental illness [26,27,28], and that this makes people with mental illness less likely to seek professional help and then hinders their recovery [29]. Furthermore, health professionals and students may avoid disclosing their own mental health problems and may be reluctant to seek help [30,31,32,33,34]. Untreated mental health problems may negatively influence the quality of care they provide, resulting in problems for patients, health services, and society [35].

Various scale-based measures have been developed for MHL, including the Mental Health Literacy Scale [36], the Mental Health Literacy Measure [37], and the Mental Health Literacy Questionnaire for young people [38] and young adults [39]. However, these tools are designed for the general public, and as yet there is no appropriate measure to assess the MHL levels of health professionals and students. Most measurement tools currently used to evaluate MHL for members of the public tend to focus more on aspects of mental ill-health (such as the recognition of mental illness) rather than on mental health promotion (such as the maintenance of mental health) [36,37,39]. In addition, apart from the MHL Scale, the MHL measures do not include items that assess the stigma of mental illness. It is noteworthy that the MHL Questionnaire includes an item on the erroneous perception of mental illness (e.g.; “Mental disorders don’t affect people’s behavior”) [38,39], but none on the common stigmatizing belief (e.g.; that people with mental illness are dangerous, eliciting an emotional reaction to them), as highlighted by research on mental illness stigma [40]. Finally, the current MHL measures include items regarding either a help-seeking attitude [36,38,39] or help-seeking efficacy [37], although both of these are the critical determinants of help-seeking behaviors.

To address these issues, we constructed and evaluated a scale to assess the MHL of health professionals and students. This addresses five critical components of MHL: (1) understanding how to obtain and maintain good mental health, (2) understanding mental disorders and their treatment, (3) addressing stigmatized attitudes related to mental disorders, (4) enhancing help-seeking efficacy, and (5) enhancing help-seeking attitudes.

## 2. Methods

### 2.1. Initial Construction of the Scale

The first phase of constructing the Mental Health Literacy Scale for Healthcare Students (MHLS-HS) involved a review of previous research about the measurement of MHL. This generated 94 items pertaining to the five components of MHL. This item pool referenced items from the following sources: the Mental Health Promoting Knowledge Measure [41], the MHL Scale [36], the Mental Health Literacy Measure [37], the Mental Health Knowledge Questionnaire [42], the Community Attitude Towards the Mentally Ill Scale (Chinese version) [43], the Endorsed and Anticipated Stigma Inventory [44], the Opening Minds Stigma Scale for Health Care Providers [45], the Attribution Questionnaire [46], the Attitudes Toward Seeking Professional Psychological Help Scale [47], and the Inventory of Attitudes Toward Seeking Mental Health Services [48]. All the items were translated into Chinese by the authors (H.J.C.; I.C.T.; H.S.H.) and reviewed by a clinical panel including one psychiatrist and two psychologists.

**Figure 1:**
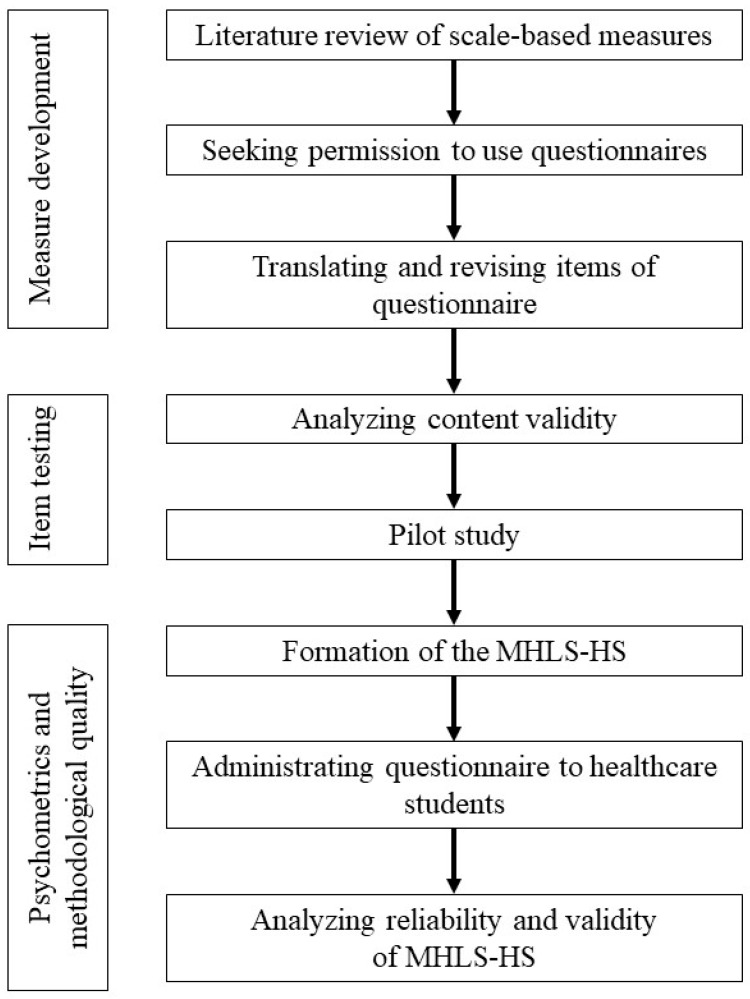
Flowchart for the development of the MHLS.

The focus group consisted of nine health professionals from a variety of healthcare disciplines (i.e., psychiatry, clinical psychology, nursing, social work, occupational therapy, and public health). These health professionals scored the items according to the importance, suitability, and clarity of each item. The content validity of the items was tested in the focus group, accordingly. A content validity index was calculated for each item, and the items for which the index was <0.80 were revised or removed [49,50]. From this, a 46-item pilot version questionnaire was generated. The flowchart for the development of the MHLS is shown in Figure 1. 

### 2.2. Pilot Study

In the second phase of the development, the pilot version questionnaire was administered to 23 medical students (mean ± SD age, 23.78 ± 1.73 years; 61% men) and 10 public health students (mean age, 22.00 ± 0.67 years; 50% men) from two universities in northern Taiwan. Based on the results of the preliminary item analysis, feedback from the participants, and the suggestions of the professionals in the focus group, a 49-item test version of the MHL scale was produced.

### 2.3. Sample

The test version was administered to students older than 20 years at 11 universities with departments of medicine and public health in Taiwan between April and June 2018. We invited 1685 individuals to participate in this study. A total of 354 respondents refused to participate and 37 were excluded after a data quality check because of their repeated endorsement of the same response option throughout the scale. Finally, 1294 respondents (response rate = 76.80%) accepted our invitation. Ethical approval for this study was obtained from the committee of the institutional review board at National Taiwan Normal University (ID: NTNUREC- 201708HS006).

### 2.4. Measures

The test version of the MHLS-HS comprised 49 items, of which 13 were reverse-scored. All the answers were evaluated on a 5-point Likert scale ranging from 1 (strongly disagree) to 5 (strongly agree). Scores for the scale were determined by summing the individual item scores. As part of the analysis, subsets of the scores for the scale were compared with those for three existing scales, as follows.

The revised five-item Positive Mental Health Scale is a short, unidimensional scale for assessing positive mental health [51]. It has demonstrated high internal consistency (α = 0.93), good retest reliability, good convergent and discriminant validity, and sensitivity to therapeutic change [51]. All the items used a 5-point Likert rating scheme, from 1 (strongly disagree) to 5 (strongly agree). Higher scores indicated greater levels of positive mental health.

The revised five-item Social Distance Scale is a measure to assess the level of desire for social distance for individuals with mental illness. The original version of the scale showed good internal consistency reliability (α = 0.75) [52]. The scale asks questions related to social distance, such as the willingness of respondents to live with a person with mental illness, have a person with mental illness marry into the family, make friends with a person with mental illness, start working closely with a person with mental illness, and introduce a job to a person with mental illness. Responses to the Social Distance Scale range from 1 (strongly disagree) to 5 (strongly agree), with higher scores indicating greater levels of desire for social distance from people with mental illness [53].

The revised Level of Contact Report is a measure to assess the respondent’s familiarity with mental illness. It lists eight situations of varying degrees of intimacy that involve people with mental illness. These range from low intimacy (“I have never observed a person that I was aware had a mental illness”) to medium intimacy (“I have a friend with a mental illness”) to high intimacy (“I have a mental illness”) [54]. The interrater reliability and validity of the measure have been confirmed by previous studies [55,56]. Scores for the revised version range from 0 to 7, with higher scores showing greater familiarity with mental illness. The index of familiarity used in the present study was the rank score of the most intimate situation indicated by the participant [54].

### 2.5. Statistical Analysis

The psychometric properties of the test version of the MHLS-HS were established by assessing its internal consistency reliability and construct validity, including the factorial validity, convergent validity, discriminant validity, and known groups validity. AMOS version 21 (IBM SPSS, Chicago, IL, USA) [57] was used for the confirmatory factor analysis to confirm the factorial validity, and SPSS version 23 (IBM Corp., Armonk, NY, USA) [58] was used for the other statistical analyses. The item analysis included an examination of item means and standard deviations. Items with extreme means (i.e., the mean value was distant from the median value) [59] and low standard deviations (less than one-sixth of the range for the responses) [60] were considered for the initial elimination.

To examine factorial validity, the participants (*N* = 1294) were randomly divided into two subsamples (each *n* = 648). Using data from the first subsample, we explored the factor structure by performing a principal axis factor analysis on the 49 items using direct oblimin rotation, deleting the items with loadings <0.40 or cross-loading [61]. We then performed univariate and multivariate normality tests for the scores for the items. Univariate normality was determined by the absolute values of skewness and kurtosis being less than 2 and 7, respectively [62], and multivariate normality was established by Mardia’s coefficient, following the method of Bollen [63]; according to this, multivariate normality is shown when Mardia’s coefficient is less than *p* (*p* + 2), where *p* is the number of observed variables.

The factor structure revealed by this exploratory factor analysis was then validated and modified by a confirmatory factor analysis, using the data for the second subsample. Any problematic items shown by the modification indices were deleted. The confirmatory factor analysis model was modified until most of the model fit indices that met the criteria. The goodness of fit of the model was assessed using absolute fit indices (the likelihood ratio (χ^2^/*df*), goodness of fit index, root mean square error of approximation, and standardized root mean square residual) and incremental fit indices (the comparative fit index and Tucker–Lewis index).

Cronbach’s alpha was used to examine the internal consistency reliability of the MHLS-HS, with α-values in the range 0.70 to 0.90, indicative of good internal consistency and considered acceptable [59]. Convergent validity was determined through composite reliability (≥0.60 was considered acceptable) [64], standardized factor loading (≥0.50 was considered acceptable) [65], and average variance extraction (≥0.50 was considered acceptable) [66]. Because people with a higher MHLS-HS exhibit lower social distance [67,68] and are more likely to take action to benefit their own mental health [3], convergent validity was tested, with additional correlation analyses for the relationship between scores in the MHL scale and scores for the Social Distance Scale [53] or Positive Mental Health Scale [51]. Discriminant validity was established by calculating the square root of the average variance extraction of each construct, which should exceed the correlation coefficient for that construct and other constructs [65].

Individuals with higher familiarity with mental illness have higher MHLS-HS than those with lower familiarity [54,55]. Accordingly, an independent sample *t* test was used to examine the known group validity by comparing the MHLS-HS scores between people with higher familiarity with mental illness (scores of 4–6 in the Level of Contact Report) and those with lower familiarity with mental illness (scores 0–3).

## 3. Results

### 3.1. Item Analysis

The item analysis for the 49 items of the test version of the MHLS-HS showed that two of the items had extreme means and low standard deviations; these were eliminated. Exploratory and confirmatory factor analyses were then performed based on the scores for the remaining 47 items of the two subsamples of participants.

### 3.2. Factorial Validity and Internal Consistency Reliability

The initial exploratory factor analysis showed that nine factors had eigenvalues >1, and the scree plot suggested a five-factor structure. An examination of the factor structure of models with five to nine factors suggested that a five-factor model produced the most theoretically meaningful factor structure. No item with cross-loading was identified, but 15 items with a loading of less than 0.40 were removed. The result was a 32-item scale based on five factors: Maintenance of Positive Mental Health (13 items), Recognition of Mental Illness (4 items), Attitude to Mental Illness Stigma (8 items), Help-Seeking Efficacy (3 items), and Help-Seeking Attitude (4 items). This accounted for 43.7% of the variance, with eigenvalues for the factors of 5.9, 3.2, 2.1, 1.5, and 1.2, respectively (Table 1). The factors explained 18.6%, 10.1%, 6.6%, 4.6%, and 3.8% of the total variance, respectively.

**Figure 2:**
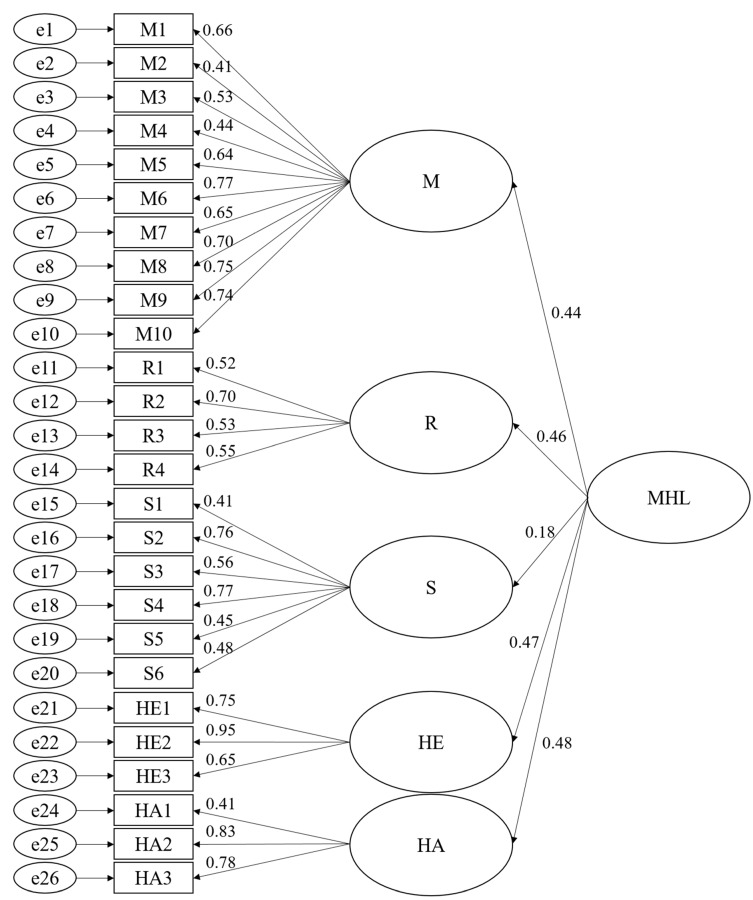
Final model of the MHL scale. M = maintenance of positive mental health; R = recognition of mental illness; S = mental illness stigma attitude; HE = help-seeking efficacy; HA = help-seeking attitude.

The assessment of normality confirmed univariate normality, with absolute value of skewness <2 and kurtosis <7. Mardia’s coefficient was 63.92; given that *p* = 32, this was less than the value for *p* (*p* + 2) = 1088, confirming multivariate normality. We then fitted a hierarchical model in which we expected that the total MHLS-HS score would be a higher order factor that influences the scores for the five factors. The model fit indices assessed with the recommended cut-off values were as follows: The likelihood ratio (χ^2^/*df*) should be 1–3 [65]; the goodness of fit index, comparative fit index, and Tucker–Lewis index should be >0.90; the root mean square error of approximation values should be <0.08 (preferably <0.05) [69]; and the standardized root mean square residual should be <0.08 [70]. Because of the high sensitivity to sample size, the chi-square value was not considered as a good index for this study. The initial second-order 32-item confirmatory factor analysis showed that only the root mean square error of approximation and standardized root mean square residual met these criteria (Table 2). This indicated that the confirmatory factor analysis model needed modification. Inspection of the modification indices indicated that six items covaried with several other items with modification indices >50 [71], so these six items were deleted sequentially. All the model fit indices were acceptable (Table 2), demonstrating that the modified second-order model with 26 items was psychometrically and statistically sound and theoretically coherent. The final 26-item five-factor model for the MHLS-HS is shown in Figure 2.

The reliability analysis demonstrated that the MHLS-HS scale had good internal reliability. Cronbach’s alpha coefficients for the five factors ranged from 0.70 to 0.87, with an overall α-value of 0.81.

### 3.3. Convergent, Discriminant, and Known Groups Validity

Most of the standardized factor loadings significantly exceeded the threshold of 0.50 (Table 3). Although only the average variance extraction of the Help-Seeking Efficacy subscale was >0.50, the composite reliability of the entire construct was >0.60, indicating that the convergent validity of the construct was adequate [66]. The results of the correlation analysis also supported the convergent validity of the MHL-HS, showing that higher total scores for the MHL-HS scale was significantly associated with lower social distance toward people with mental illness (*r* = −0.26; *p* < 0.001) and with greater positive mental health (*r* = 0.35; *p* < 0.001), as assessed by the Social Distance Scale and Positive Mental Health Scale, respectively. Stronger correlations with these two scales were observed for the Attitude to Mental Illness Stigma subscale (*r* = −0.44; *p* < 0.001) and the Maintenance of Positive Mental Health subscale (*r* = 0.36; *p* < 0.001), respectively.

As shown in Table 4, the square roots of the average variance extraction of each construct (the values of the diagonal elements in the table) were all greater than the correlation coefficients of each construct (the values below the diagonal). This confirmed the discriminant validity of the MHLS-HS.

As expected, the results of the independent sample *t* test revealed that the participants with higher familiarity with mental illness, as measured by the Level of Contact Report, scored significantly higher on the MHL-HS scale than those with lower familiarity with mental illness (mean 101.42 ± 8.86 vs. 100.01 ± 9.03; *t* (1250) = 2.79, *p* = 0.005; *d* = 0.16). Although the effect size was low, this significant result supported the known groups validity of the MHLS-HS.

## 4. Discussion

The aim of this study was to develop a scale to assess the MHL of health professionals and students and to evaluate the psychometric properties of the scale. To the best of our knowledge, the resulting MHLS-HS is the first scale-based measure specifically for assessing the MHL of health professionals and students. The scale appropriately captures the multidimensionality of MHLS-HS, with the analyses confirming the appropriateness of the five-factor structure. The final 26-item scale showed good internal consistency reliability and satisfactory construct validity, based on the analyses of factorial, convergent, discriminant, and known groups validity.

MHL is an evolving concept. In addition to the factors listed in a recent definition of MHL [1], the MHLS-HS also included the concept of a help-seeking attitude, which is a strong determinant of help-seeking behavior [10,11,12]. The 26-item MHLS-HS comprises five subscales, which correspond to the critical components of MHL. They highlight mental health promotion and aspects of mental ill-health, as well as aspects of help-seeking determinants.

The Maintenance of Positive Mental Health subscale includes items that can improve mental health, including the three basic psychological needs (competence, autonomy, and relatedness) in self-determination theory [72] and resilience [73]. According to Ryan et al. [74], meeting the three fundamental psychological needs leads to improved mental health, such as less depression and anxiety and a higher quality of life, thereby producing a positive effect on the quality of care a health professional can provide. Resilience not only protects mental well-being [75,76], but also helps to prevent cognitive errors, enabling health professionals to care for patients safely and effectively [77,78].

The Recognition of Mental Illness subscale includes items about the recognition of the most severe and common mental illnesses (schizophrenia, anxiety disorders, depression, and substance-related and addictive disorders), which can result in disability and a substantial decrease in quality of life in those with mental illness [79]. Many mental disorders remain undiagnosed in clinical practice [21,23], so enhancing the ability of health professionals and students to recognize mental illnesses during healthcare service delivery may promote their early detection and appropriate treatment, thereby improving the health outcomes of the individuals with mental illness.

The Attitude to Mental Illness Stigma subscale includes reverse-scored items that measure concepts of stigma that have been commonly highlighted by previous research, including public stigma (i.e., belief that others perceive an individual as socially unacceptable), dangerousness (i.e., belief that the individual with mental illness is dangerous) [80], and emotional reaction (i.e., fear of individuals with mental illness) [81]. According to the social attribution theory, perceived dangerousness may induce an emotional response such as fear, increasing discriminatory behavior, such as social distance [46]. Any stigmatizing attitudes among health professionals and students toward individuals with mental illness may have a negative effect on the recovery of those patients [29]. Detecting stigmatizing attitudes among health professionals and students may therefore provide information that helps researchers to tailor anti-stigma education interventions for this group.

The Help-Seeking Efficacy subscale includes items pertaining to knowing when and where to seek help and developing competencies designed to improve an individual’s own mental health care and self-management capabilities, as proposed by [1]. In general, help-seeking efficacy has been considered to have a moderating effect on help-seeking intention and behavior [82]. The Help-Seeking Attitude subscale comprises items about attitudes toward seeking professional help for an individual’s own psychological difficulties [47]; such attitudes have been reported to be a strong predictor of help-seeking intention and behavior [10,11,12]. The promotion of help-seeking for mental illness is essential for MHL research [83].

Scores in the MHLS-HS showed significant correlations with scores for the Social Distance Scale and Positive Mental Health Scale. These findings are consistent with the results of previous studies that reported that individuals with greater levels of MHL have significantly lower social distance toward people with mental illness [67,68] and more positive mental health [3]. We hypothesized that a group with higher scores on the revised Level of Contact Report would have significantly higher scores on the MHLS-HS. This hypothesis was fully supported by the results of the present study. In line with previous studies, those with higher familiarity with mental illness, as measured by the Level of Contact Report, had significantly higher overall scores for the MHL than their counterparts [36,37,84]. However, in contrast to our findings, previous studies have reported that contact experience was associated with negative results [85]. Thus, the key ingredients of contact (emphasizing and demonstrating recovery as both real and probable and communicating the message that health professionals and students play a vital role in recovery) should be highlighted in mental interventions for health professionals and students [86].

This study had several limitations. First, the participants were limited to medical and public health students, although we attempted to recruit clerks from the hospital. We invited health professionals from a variety of healthcare disciplines to confirm the content validity of the pilot version of the scale and subsequently revised the scale according to their feedback; nevertheless, generalization of the findings to health professionals and students in other disciplines may be restricted. Second, the scale is a self-report instrument. Although anonymity and confidentiality were assured in our study, this study may not have been entirely free of social desirability bias, especially for the measure of attitudes to stigma toward mental illness. Third, the study did not examine the test–retest reliability of the scale.

Future studies are needed to examine whether the findings of this study can be replicated for other health professional groups. A test–retest reliability analysis of the scale is needed to examine its temporal stability. The analysis should include the scale of actual help-seeking behavior and should further examine the predictive validity of the two help-seeking-related subscales for actual help-seeking behavior. The MHLS-HS can be used to investigate the MHL levels of medical and public health students and can be used to provide a quick comprehensive overview of the current condition of MHL among healthcare students. The results of this study suggest that the MHLS-HS can also serve as an instrument to evaluate the effectiveness of interventions designed to promote the MHL of medical and public health students.

## 5. Conclusions

In conclusion, the 26-item MHLS-HS developed in this study is a valid and reliable five-dimensional scale with an adequate model fit. It is the first scale-based measure specifically designed to assess the MHL of healthcare students, and it covers both mental health promotion and aspects of mental ill-health. It has the potential to complement and expand current measures of MHL, providing a quick and comprehensive overview of the current condition of MHL. As such, MHLS-HS can be used to identify mental health literacy gaps in medical and public health students and to evaluate the impact of remedial curricular interventions.

## Figures and Tables

**Table 1:** Exploratory factor analysis loadings for the 32-item mental health literacy (MHL) scale (direct oblimin rotation) (n = 648).

Item	Factor Loading				
	F1	F2	F3	F4	F5
Maintenance of positive mental health					
Handling stressful situations in an appropriate manner.	0.74				
Mastering your own negative thoughts.	0.67				
Making decisions based on your own will.	0.48				
Setting limits for what is acceptable for you.	0.50				
Setting limits for your own actions.	0.59				
Having religious or spiritual beliefs.	0.41				
Feeling that you belong in a group (such as a community, workplace, or school)	0.63				
Feeling valuable irrespective of your own accomplishments.	0.65				
Able to adapt to change.	0.68				
Able to achieve goals despite obstacles.	0.68				
Able to stay focused under pressure.	0.72				
Not easily discouraged by failure.	0.77				
Able to handle unpleasant feelings.	0.73				
Recognition of mental illness					
If someone experiences excessive worry about events or activities where this level of concern was not warranted, has difficulty controlling this worry, and has physical symptoms, such as muscle tension and feelings of fatigue, then to what extent do you think it is likely he or she has an anxiety disorder?		0.54			
If someone experiences a low mood for two or more weeks, with a loss of pleasure or interest in their normal activities, and changes in their appetite and sleep patterns, then to what extent do you think it is likely he or she has a depressive disorder?		0.65			
If someone requires higher doses of a drug to get the same effect, then to what extent do you think it is likely that he or she has a substance-related addictive disorder.		0.64			
If someone experiences delusions and hallucinations, and talks to him- or herself, then to what extent do you think it is likely that he or she has schizophrenia?		0.66			
Attitude to mental illness stigma					
I think people with mental illness are burdens to society. (R)			0.56		
I think having a mental illness is shameful. (R)			0.55		
Most people with mental illness are dangerous. (R)			0.64		
Most people with mental illness are at risk for self-harm. (R)			0.43		
Most people with mental illness may pose a risk to the public. (R)			0.69		
I think people with mental illness are unpredictable. (R)			0.46		
I think people with mental illness are irritating. (R)			0.70		
I think people with mental illness are frightening. (R)			0.71		
Help-seeking efficacy					
I know where to go to receive mental health promotion services.				0.71	
I know where to go to receive psychiatry services.				0.90	
I know where to seek information about mental illness (e.g.; family doctor, internet, or friends).				0.65	
Help-seeking attitude					
To address a mental health problem, my first choice would be to seek help from mental health professionals.					0.57
I trust healthcare organizations to provide mental health services.					0.58
If I face emotional problems, I would seek help from mental health professionals.					0.80
If I were having a mental breakdown, my first inclination would be to seek the attention of a mental health professional.					0.77
Eigenvalue	5.94	3.22	2.12	1.46	1.22
% of explained variance	18.58	10.07	6.64	4.57	3.82
Total variance explained	43.68				

R = reversed-scored items. Factor loadings >0.40 are shown.

**Table 2:** Fit indices for CFA models. Second-order 5-factor solutions for the MHL scale.

Global Model Fit	Acceptable Criteria	Initial Second-Order Model (32 Items)	Modified Second-Order Model (26 Items)
Absolute fit indices			
χ^2^	*p* > 0.05	1682.15 (*p* < 0.001)	733.78 (*p* < 0.001)
χ^2^/*df*	1 < χ^2^/*df* < 3	3.67	2.50
GFI	>0.90	0.85	0.92
RMSEA	<0.08	0.06	0.048
SRMR	<0.08	0.07	0.06
Incremental fit indices			
CFI	>0.90	0.83	0.91
TLI	>0.90	0.81	0.90

Confirmatory factor analysis (CFA); Goodness of fit index (GFI); root mean square error of approximation (RMSEA); standardized root mean square residual (SRMR); comparative fit index (CFI); Tucker–Lewis Index (TLI); degree of freedom (*df*).

**Table 3:** Factor loading, convergent reliability, and convergent validity of confirmatory factor analysis.

Factor	Item	λ	SMC	SE	t	CR	AVE
M	M1	0.66	0.44	-	-	0.87	0.41
	M2	0.41	0.17	0.06	9.55 ***		
	M3	0.53	0.28	0.07	12.22 ***		
	M4	0.44	0.20	0.08	10.30 ***		
	M5	0.64	0.41	0.08	14.44 ***		
	M6	0.77	0.59	0.05	16.82 ***		
	M7	0.65	0.42	0.07	14.54 ***		
	M8	0.70	0.49	0.07	15.60 ***		
	M9	0.75	0.57	0.08	16.50 ***		
	M10	0.74	0.55	0.06	16.29 ***		
R	R1	0.52	0.27	-	-	0.67	0.34
	R2	0.71	0.50	0.14	9.37 ***		
	R3	0.53	0.29	0.14	8.68 ***		
	R4	0.55	0.30	0.12	8.79 ***		
S	S1	0.41	0.17	-	-	0.75	0.35
	S2	0.76	0.58	0.22	9.21 ***		
	S3	0.57	0.32	0.20	8.37 ***		
	S4	0.77	0.59	0.22	9.22 ***		
	S5	0.45	0.20	0.17	7.50 ***		
	S6	0.48	0.23	0.17	7.73 ***		
HE	HE1	0.75	0.57	-	-	0.83	0.63
	HE2	0.95	0.89	0.06	18.35 ***		
	HE3	0.65	0.42	0.04	16.45 ***		
HA	HA1	0.41	0.17	-	-	0.73	0.49
	HA2	0.83	0.70	0.28	8.88 ***		
	HA3	0.78	0.60	0.27	9.14 ***		

M = maintenance of positive mental health; R = recognition of mental illness; S = mental illness stigma attitude; HE = help-seeking efficacy; HA = help-seeking attitude; λ = Standardized factor loading. Square multiple correlation (SMC); standard error of factor analysis (SE); composite reliability (CR); average variance (AVE) *** *p* < 0.001.

**Table 4:** Inter-subscale correlation and discriminant validity of the MHLS-HS scale.

Subscale	M	R	S	HE	HA
M	(0.64)	-	-	-	-
R	0.25 ***	(0.58)	-	-	-
S	0.05	0.001	(0.59)	-	-
HE	0.16 ***	0.15 ***	0.20 ***	(0.79)	-
HA	0.16 ***	0.14 ***	0.002	0.26 ***	(0.70)

M = maintenance of positive mental health; R = recognition of mental illness; S = mental illness stigma attitude; HE = help-seeking efficacy; HA = help-seeking attitude. Diagonal elements are the square roots of the average variance (AVE) of each construct. *** *p* < 0.001.
